# Stimulation of MMP-11 (stromelysin-3) expression in mouse fibroblasts by cytokines, collagen and co-culture with human breast cancer cell lines

**DOI:** 10.1186/1471-2407-4-40

**Published:** 2004-07-25

**Authors:** Saxon Selvey, Larisa M Haupt, Erik W Thompson, Klaus I Matthaei, Michael G Irving, Lyn R Griffiths

**Affiliations:** 1 Genomics Research Center, School of Heath Science, Griffith University, Gold Coast, Australia; 2 Stem Cell and Tissue Repair Laboratory, Institute of Molecular and Cell Biology, Singapore; 3 VBCRC Invasion and Metastasis Unit, St. Vincent's Institute of Medical Research and Department of Surgery, University of Melbourne, Melbourne, Australia; 4 John Curtin School of Medical Research, Australian National University, Canberra, Australia; 5 Faculty of Health Science and Medicine, Bond University, Gold Coast, Australia

## Abstract

**Background:**

Matrix metalloproteinases (MMPs) are central to degradation of the extracellular matrix and basement membrane during both normal and carcinogenic tissue remodeling. MT1-MMP (MMP-14) and stromelysin-3 (MMP-11) are two members of the MMP family of proteolytic enzymes that have been specifically implicated in breast cancer progression. Expressed in stromal fibroblasts adjacent to epithelial tumour cells, the mechanism of MT1-MMP and MMP-11 induction remains unknown.

**Methods:**

To investigate possible mechanisms of induction, we examined the effects of a number of plausible regulatory agents and treatments that may physiologically influence MMP expression during tumour progression. Thus NIH3T3 and primary mouse embryonic fibroblasts (MEFs) were: a) treated with the cytokines IL-1β, IL-2, IL-6, IL-8 and TGF-β for 3, 6, 12, 24, and 48 hours; b) grown on collagens I, IV and V; c) treated with fibronectin, con-A and matrigel; and d) co-cultured with a range of HBC (human breast cancer) cell lines of varied invasive and metastatic potential.

**Results:**

Competitive quantitative RT-PCR indicated that MMP-11 expression was stimulated to a level greater than 100%, by 48 hour treatments of IL-1β, IL-2, TGF-β, fibronectin and collagen V. No other substantial changes in expression of MMP-11 or MT1-MMP in either tested fibroblast culture, under any treatment conditions, were observed.

**Conclusion:**

We have demonstrated significant MMP-11 stimulation in mouse fibroblasts using cytokines, matrix constituents and HBC cell lines, and also some inhibition of MT1-MMP. Our data suggest that the regulation of these genes in the complex stromal-epithelial interactions that occur in human breast carcinoma, is influenced by several mechanisms.

## Background

Integral to both normal and pathological tissue remodeling, the matrix metalloproteinase (MMP) family of proteolytic enzymes collectively degrades laminin, collagen, gelatin, and other protein components of the extracellular matrix [1,2]. MMP expression allows degradation of the basement membrane, an event essential to the process of tumour metastasis [3]. The induction of specific MMPs such as MT1-MMP and MMP-11 have been shown to correlate with tumour invasiveness and resultant metastasis in several human cancers, including breast carcinoma [4-7]. Thus, gene expression analysis of these two MMPs may have clinical application in breast cancer diagnosis, management and therapy.

Little is known about the mechanisms underlying MT1-MMP and MMP-11 induction. Previous studies have revealed induction of MT1-MMP in human fibroblasts grown on interstitial collagen (collagen I), which is abundant around tumours and during wound healing [8]. Furthermore, MMP-11 has been shown to be induced by retinoic acid [9]. This study aimed to investigate possible regulatory agents and treatments inducing MT1-MMP and/or MMP-11 expression. Specifically, MMP induction was measured in NIH3T3 and primary mouse embryonic fibroblasts (MEFs) subjected to each of the following potentially regulatory agents: collagen I, IV and V, concanavalin-A, fibronectin, and several exogenous cytokines. As induction by such agents could be transitory, treatment times were varied from 3, 6, 12, 24 and 48 hours, and were immediately followed by RNA extraction.

As collagen I has been reported to induce MT1-MMP expression, detection of such induction by quantitative RT-PCR would provide an ideal basis for testing further regulatory agents. Collagen IV and V appeared to be ideal candidates for further analysis of MMP induction as they are present in the basement membrane and would therefore be released specifically during early tumour invasion and metastasis, which coincides with MT1-MMP and MMP-11 expression *in vivo*. Type IV collagen has been shown to mediate pro-MMP-2 activation in HT1080 cells, a human fibrosarcoma cell line, without inducing either a transcriptional modulation of MMP-2 or MT1-MMP expression nor any alteration of MT1-MMP protein synthesis or processing [10]. Concanavalin-A is a plant lectin reported to stimulate activation of MMP-2 [11,12]. Fibronectin encourages cell adhesion and HT1080 cells cultured on fibronectin have exhibited elevated levels of MT1-MMP protein [13-15].

We investigated the potential role of the following cytokines, interleukin (IL)-1β, IL-2, IL-6, IL-8 and transforming growth factor (TGF)-β in MT1-MMP and MMP-11 regulation. All of these cytokines are reportedly released by neoplastic cells and are capable of affecting transcription in fibroblasts [16-19]. IL-2 is also released by tumour infiltrating lymphocytes (TILs) and IL-6 is released by tumour associated macrophages (TAMs), TILs and fibroblasts [16,20-22]. Moreover, MT1-MMP and MMP-11 have both been reported to be induced in human fibroblasts treated with conditioned medium from MDA-MB-231, a highly invasive mammary tumour cell line [23]. Thus, we investigated the induction of MT1-MMP and MMP-11 in NIH3T3 and MEFs co-cultured with a range of HBC (human breast cancer) cell lines of varied invasive and metastatic potential. This allowed direct *in situ *interaction of MMPs, growth factors and other potentially regulatory agents between the fibroblasts and HBC cells.

## Methods

### Cell lines

NIH3T3 culture was obtained from the ATCC, while MEF cultures were constructed at the John Curtin School of Medical Research, Australian National University. Briefly, embryos were removed from 13-day pregnant mice. Following removal of the liver and head, the remaining tissue was placed in PBS (1× pH 7.4, Sigma-Aldrich). Tissue was homogenised using an 18 guage syringe (Terumo) in PBS, plated in 25 cm^2 ^tissue culture flasks (Costar) DMEM (Dulbecco's modified Eagles medium) with 10% FCS (Invitrogen) and allowed to attach for 6–12 hrs at 37°C, 5% CO_2 _prior to use. HBC cell lines were originally obtained from the ATCC and maintained by The Lombardi Cancer Center. ML-20 cells were derived from The Lombardi Cancer Center MCF-7 cells by clonal selection after transfection with a CMV-driven expression plasmid encoding bacterial β-galactosidase.

### Collagen coats and fibronectin treatment

Collagen coats and fibronectin treatments were prepared on non-coated tissue culture plates as follows: prior to seeding, triplicate sets of 2 ml 24 well plates (Costar) were overlaid with 150 μl of collagen I, IV and V (Sigma, 1 mg/ml) solutions. For the fibronectin treatments, 15 μg of fibronectin (Promega), equivalent to 5 μg/cm^2 ^of flask area were added to each well in 2 ml 24 well plates (Costar). The wells were then incubated at 37°C for 1 hour to allow the collagen coats and fibronectin to set in the wells. Excess solution was aspirated from all wells before seeding with 10^5 ^cells from either NIH3T3, or MEF cultures in 1 ml DMEM - 10% fetal calf serum (Invitrogen). Treated cultures were then incubated at two time-points, 24 hours and 48 hours at 37°C, 5% CO_2_, before undergoing RNA extraction.

### Con-A treatment

Non-coated 2 ml 24 well tissue culture plates (Costar) were seeded with either NIH3T3 or MEF at 10^5 ^cells in 1 mL Dulbecco's modified Eagles medium (DMEM) - 10% fetal calf serum (Invitrogen). Treatments consisted of 25 μg Con-A added to the 1 ml of DMEM in each well. Treated cultures were then incubated at two time-points, 24 hours and 48 hours at 37°C, 5% CO_2_, before undergoing RNA extraction.

### Cytokine treatments

2 ml 24 well plates (Costar) were seeded with 10^5 ^cells from either NIH3T3 or primary mouse fibroblast cultures (passage 5) in 1 mL Dulbecco's modified Eagles medium (DMEM) - 10% fetal calf serum (Invitrogen). Cultures were incubated at 37°C, 5% CO_2 _for 24 hours prior to cytokine treatment, thus allowing complete attachment. All treatments were separate and consisted of final solutions of 100 units/ml IL-1β, IL-2, IL-6, 0.1 units/ml of IL-8 and 1 ng/ml pan-TGF-β (Sigma-Aldrich) in cultures grown in triplicate.

### Co-cultures

30 mm 6 well plates (Costar) were seeded with 10^6 ^cells of either NIH3T3 or MEFs (passage 5), which were each allowed to attach overnight in 1 mL Dulbecco's modified Eagles medium (DMEM) - 10% fetal calf serum (Invitrogen). The DMEM was then aspirated and 24 mm Transwell Porous Cell Culture Inserts (Costar) were seeded with 10^5 ^cells of either MDA-MB-231, MDA-MB-435, MDA-MB-436, MCF-7s or ML-20 epithelial tumour cultures in 2 ml of RPMI medium (Trace Biosystems) - 10% fetal calf serum. Use of the porous inserts meant that co-cultures were not in direct physical contact, but shared growth media at all times. These HBC cultures exhibit different levels of invasive and metastatic potential (see Table 1). Each co-culture then underwent 48 hour incubation at 37°C, 5% CO_2_, before RNA extraction.

### RNA extraction and quantification

Total RNA was extracted from the fibroblasts using an RNeasy Mini Kit (QIAGEN) as previously described [24]. Total RNA was then quantified at OD_260 _using a QuantaGene spectrophotometer (The Australian Chromatography Company, Sydney, NSW, Australia).

### Oligonucleotide primers

All 3' primers were labeled with the TET dye phosphoramidite and were commercially obtained (Applied Biosystems, Brisbane, QLD, Australia). Oligonucleotides for the target genes, MT1-MMP and MMP-11, were generated using Amplify 2.1 analysis software in conjunction with published cDNA sequences [6,25]. The oligonucleotide sequences for the housekeeping gene 18S rRNA were supplied by Ambion. All sequences utilised are outlined in Table 2.

### Constructing cDNA competitive templates

cDNA competitive templates for the target genes, MT1-MMP and MMP-11 were made by PCR amplification using purpose built 47-mer oligonucleotides as described [26]. The 47-mers consisted of a 23 bp 5' primer complementary to target sequence either 28 bp, for MT1-MMP, or 20 bp, for MMP-11, further 5' than the remaining 24 bp of the 47-mer, which consisted of the normal 5' primer sequence for PCR. Hence, a PCR product generated from these 5' 47-mers in combination with the normal 3' primers, produced fragments shorter than when using the normal 5' primer, as these deletions were left out during sequence extension complementary to the 47-mer. Both 5' and 3' ends of the competitive template remain complementary to normal primers and therefore undergo further complementary-copying during additional PCR in a manner identical to the target sequences, except that the missing nucleotide fragments remain absent. (Table 2)

### Constructing mRNA competitive templates

The cDNA competitive templates generated by PCR with the 47-mers described above were each ligated into pGem-T Easy Vectors (Promega, Annandale, NSW, Australia). JM109 (Promega) competent cells were then transformed and grown overnight. Plasmid DNA was extracted using a Magic Miniprep kit (Promega) and linearised by nuclease digest. Mimic RNA was generated from the "mimic" plasmids by transcription of the insert with T7 RNA polymerase (Promega). The post-transcription mixture then underwent RQ1 DNase (Promega) digestion; the remaining RNA was then purified using QIAGEN RNeasy Minikit, RNA competitive template was then resuspended in DEPC-treated H_2_O and stored at -70°C. To ensure that amplification would not occur as a result of residual DNA contamination, control samples were run in triplicate without the reverse transcriptase thermal cycle. No amplification products were visualised on resultant agarose gels.

### RT-PCR

Single tube, one step RT-PCR was performed as previously described [24] for all samples using an ABI 480 thermal cycler in 25 μl volume containing: 24 ng total RNA, 1–2 μl of RNA competitive template, being equivalent to 24 ng of control total RNA, 200 mM dNTPs (Promega), 5× RT-PCR buffer [300 mM Tris-HCl, pH 8.3; 2.5 mM DTT; 250 mM KCl; 0.5% Triton X-100 (Evergreen Scientific); 30 μm EDTA; 7.5 mM MgCl_2_], 0.4 μM each primer, 1 unit Taq polymerase (Perkin Elmer ABI), 1.2 units AMV reverse transcriptase (Promega) and 900 ng tRNA (Boehringer Mannheim)]. Thermal cycling was as follows: 30 mins 50°C (reverse transcription), then 2 mins 95°C, followed by 35 cycles of 30 sec 95°C, 30 sec 65°C, 45 sec 72°C, followed by a final incubation of 5 mins at 72°C.

### GeneScan

3' primers for MT1-MMP, MMP-11 and 18S rRNA were labeled with TET fluorescent dye, thus allowing RT-PCR samples to be analysed by capillary electrophoresis using a 310 Genetic Analyzer with GeneScan software (ABI) as described previously [24]. Using laser technology to excite the fluorescently labeled primers, samples were sized to within one base pair and the amount of amplification product of any given size, determined by fluorescence peak area.

### Ratio calculations

The GeneScan values for fluorescence peak area of the target gene transcripts and the control transcripts produced the target:control ratio. All graphed values represent averages from triplicate samples and were adjusted to produce a control value of 1.00. These target:control ratio values were used to plot time series graphs of all combined data for each gene. Data on the time series graphs was then normalised to 18S rRNA expression, as measured in the same respective cultures. The 0.5× control and 2.0× control are the resultant target:control ratios produced using half and double the amount of total RNA used in the control sample, respectively.

## Results

### Induction treatments

#### MT1-MMP: 3, 6, 12 and 24 hour treatments

Competitive quantitative RT-PCR using capillary electrophoresis and GeneScan analysis revealed no substantial changes in expression of MT1-MMP in either NIH3T3, or MEF cultures under any treatment conditions carried out over 3, 6, 12, and 24 hours (Tables 3 and 4). Although as indicated in Table 3 there are some minor initial variations, when the normalised to 18S rRNA no substantial changes are observed. The data presented are derived from GeneScan values for fluorescence peak area of the target gene transcripts and the control transcripts, thus producing the target:control ratio. All values represent averages from samples run in triplicate and have been adjusted to produce a control value of 1.00. Results for these time periods display only minor variations and generally fall between 0.5× control and 2.0× control expression levels.

#### MT1-MMP: 48 hour treatments

##### Induction

Initial analyses of the 48 hour IL-1β, IL-2 and fibronectin treatments indicated consistently basal MT1-MMP expression levels (Table 5). Yet data for these treatments do indicate induction when normalized to the unexpectedly low level of 18S rRNA expression (Tables 7 and 8). However, 18S rRNA expression is monitored primarily to double check for substantial anomalies in mRNA quantification when positive results are found, rather than as a precise means of normalization for results which appear consistent. Furthermore, competitive RT-PCR of MT1-MMP may be considered more accurate than non-competitive differential RT-PCR of 18S rRNA. Initial competitive RT-PCR of MT1-MMP expression did produce consistently unchanged results for these treatments, while expression of 18S rRNA was found to display a relatively high level of variation in this treatment group. Specifically, the level of 18S rRNA expression exhibited in the control was high, therefore causing all other treatments to appear relatively low, which in turn caused an artificial elevation of all treatment data values during normalization. Hence, a consistent group result for competitive RT-PCR of MT1-MMP should not be discarded in favour of a single set of unusually efficient differential RT-PCRs of 18S rRNA control samples. In our opinion, MT1-MMP expression should be considered unaffected by IL-1β, IL-2 and fibronectin treatments during this time schedule.

##### Inhibition

Results indicated substantial inhibition of MT1-MMP expression by collagen I and collagen IV (Tables 5 and 6). Expression values for these treatments remained well below the 0.5× control expression level even after normalization (Tables 7 and 8), despite this procedure raising the values in a way that may have been to some degree artificial, as described above.

#### MMP-11: 3, 6, 12 and 24 hour treatments

Competitive quantitative RT-PCR using capillary electrophoresis and GeneScan analysis revealed no substantial changes in expression of MMP-11 in either fibroblast culture under any treatment conditions carried out over 3, 6, 12 and 24 hours (Tables 3, 4 and 5). Results for these time periods display only minor variations and generally fall between 0.5× control and 2.0× control expression levels. The use of an internal competitor to obtain increased specificity of target:template detection demonstrate data for these time periods and is highly consistent. Such consistency within groups of treatments carried out simultaneously (i.e: over an individual time schedule) further suggests that the minor variations from control expression levels which are present, may be due to amplification anomalies rather than individual differences in gene expression caused by specific treatments.

#### MMP-11: 48 hour treatment

At the 48 hour time point, MMP-11 expression appeared to be stimulated at least 2 fold by IL-1β, TGF-β, fibronectin and collagen V. Results indicate induction of MMP-11 by these agents independently of normalization to 18S rRNA expression (Tables 7 and 8), which further enhanced these expression values. IL-2 and IL-6 appeared to produce a very mild induction of MMP-11 without normalization to 18S rRNA, and only appeared to produce a greater than 2 fold induction when undergoing such normalization.

The data suggesting that MMP-11 expression is induced by IL-1β, TGF-β, fibronectin and collagen V is highly persuasive given that these are the only data that demonstrate considerable within-group variation. The standard deviation for the 48 hour MMP-11 treatments was 47.6 while the second greatest standard deviation value was 40.5 for the 24 hour MMP-11 treatments. The average for all other MMP-11 groups was 17.3. This increase over the average variation is suggestive of differences in initial transcript levels for individual treatments. Furthermore, the fact that MT1-MMP expression levels (non-normalised) remained consistently normal for this treatment, effectively acting as an additional unregulated control, lends further credit to the likelihood of specific regulation of MMP-11 after 48 hours of treatment. There are also mild trends toward up regulation for the IL-2 and IL-6 treated samples.

### Co-culture with HBC cell lines

Initial results demonstrate no induction or inhibition of either MT1-MMP or MMP-11 in mouse fibroblasts by co-culture with any of the HBC cell lines (Table 9). Results are highly consistent and all treatment values fall between 0.5× control and 2.0× control. Moreover, minor variations from control expression values observed for each co-culture are almost identical, between the two target genes. Hence, the mild expression elevations seen with ML-20 and MDA-MB-436 for both MT1-MMP and MMP-11 are in our opinion, artifactual.

## Discussion

This study investigated the effects of a number of possible regulatory agents on the expression of two members of the MMP gene family for their role in human breast cancer metastases. NIH3T3 and MEF cultures were treated with cytokines, collagens, fibronectin, Con-A or matrigel, and co-cultured with various HBC cell lines. The expression of MT1-MMP and MMP-11 was then determined by competitive RT-PCR using capillary electrophoresis and GeneScan technology. Only MMP-11 expression at 48 hours was affected by treatment with IL-1β, TGF-β, fibronectin and collagen V, in the tested fibroblasts cultures.

During tumour progression, the release of exogenous cytokines by neoplastic cells and immunological cells may cause a stimulatory effect on adjacent stromal fibroblasts, resulting in matrix metalloproteinase (MMP) induction. Past studies indicate that some MMPs undergo regulatory changes under the influence of cytokines [18,27]. Another recent study reported the induction of MMP-11 expression in primary cultured human fibroblasts over a 48 hour period by IL-6, IGF-2, EGF and PDGR-BB, but not by IL-1β or TNF-β [28]. Similar to the MT1-MMP results presented in the present study, MT1-MMP gene expression was reportedly not affected by insulin-like growth factor (IGF)-2, epidermal growth factor (EGF), platelet derived growth factor (PDGR)-BB, IL-6, TNF-β or IL-1β [28]. Any influence exerted on MT1-MMP by cytokines may be of particular interest because of its specific interaction with MMP-2, an MMP with a broad substrate specificity. Hence, further analysis of candidate stimulatory agents is required to elucidate possible mechanisms by which MT1-MMP expression is stimulated.

Our study did however, find evidence for an inhibitory effect from both collagen I and collagen IV on MT1-MMP expression. Curiously, there is evidence suggesting that collagen I actually stimulates MT1-MMP mRNA production in fibroblasts and activates MMP-2 in a variety of cells over a longer time period [8,29]. However, past studies on rat smooth-muscle cell culture support findings that collagen IV exhibits MT1-MMP inhibitory effects [12].

Despite reports of MDA-MB-231 conditioned media inducing MT1-MMP expression [23] in human fibroblasts, co-culture between MEFs and MDA-MB-231, did not produce induction of either MT1-MMP or MMP-11 in this study. This finding is likely to be due to species-specificity, since mouse mammary stromal cells are not capable of supporting normal human mammary reorganization *in vivo *[30].

Although the co-cultures carried out here did not allow cell-cell contact, the previous study also involved no cell-cell contact, but rather treatment with pre-conditioned media (application of isolated medium from one cell population following *in vitro *expansion to an another isolated cell population). Another possible explanation is that the basal level of expression of MT1-MMP and MMP-11 in cultured murine fibroblasts is elevated, thus rendering them refractory to exogenous regulatory agents. We are currently investigating this possibility.

## Conclusion

This study examined the effects of a number of regulatory agents and treatments on the induction of MMP-11 and MT1-MMP gene expression, factors that may influence breast cancer tumour progression. Stimulation of MMP-11 was demonstrated after treatments by both cytokines (IL-1β, TGF-β) and by matrix constituents (collagen IV and fibronectin), whilst MT1-MMP was inhibited by matrix constituents (collagen I and fibronectin). The results presented suggest that several mechanisms may be involved in MMP-11 and MT1-MMP regulation as part of the complex epithelial-stromal interactions that occur within human breast carcinomas.

## Competing interests

None declared.

## Authors contributions

S.S. performed the molecular genetic and *in vitro *studies, designed PCR primers and drafted the manuscript. L.M.H. contributed to the manuscript design and finalisation. E.W.T. contributed toward the design of the study. K.I.M. contributed toward the design and performance of competitive PCRs. M.G.I. participated in the conception and design of the study. L.R.G. participated in the conception and design of the study and its coordination. All authors read and approved the final manuscript.

## Pre-publication history

The pre-publication history for this paper can be accessed here:



## Figures and Tables

**Table 1:** Invasive and metastatic potential of HBC cell lines [31].

**Cell Line**	**Estrogen Receptor Status**	**Vimentin Expression**	**Invasiveness**
**MDA-MB-231**	-	+	+++
**MDA-MB-435**	-	+	++
**MDA-MB-436**	-	+	++
**MCF-7**	++	-	+
**NCI-adr**	-	+	++
**MCF-7stv**	+	-	+
**ML-20**	+	-	+

**Table 2:** Oligonucleode primer sequences.

**Gene**	**5' sequence**	**3' sequence**	**Standard amplicon**	**Competitor sequence (standard 5' primer plus)**	**Competitor amplicon**
**MT1-MMP**	gACAgTACACCCTTTgATggTgAA	gATCgTTAgAATgTTCCAggCCTA	197 bp	*CAgGCCCCAATATTggAggggAT*	**169 (-28 bp)**
**MMP-11**	CCTTCCAggATgCTgAgggCTAT	ATgACAgCATggTCgTTCCTACAA	330 bp	*TCTCTACTggAggTTTgATCCCgT*	**310 (-20 bp)**
**18s rRNA**	TCAAGAACgAAAgTCggAgg	ggACATCTAAgggCATCACA	490 bp	N/A	N/A

**Table 3:** MT1-MMP, MMP-11 and 18S rRNA Target:Control ratios for 3, 6 and 12 hour treatments of NIH3T3 cells.

**Treatment**	**Target:Control Ratio**								
	**3 Hours**			**6 Hours**			**12 Hours**		
	
	MT1-MMP ± SEM	MMP-11 ± SEM	18S rRNA ± SEM	MT1-MMP ± SEM	MMP-11 ± SEM	18S rRNA ± SEM	MT1-MMP ± SEM	MMP-11 ± SEM	18S rRNA ± SEM

**Control**	1.00 ± 0.08	1.00 ± 0.10	1.00 ± 0.04	1.00 ± 0.08	1.00 ± 0.10	1.00 ± 0.04	1.00 ± 0.08	1.00 ± 0.08	1.00 ± 0.02
**TGF-β**	0.49 ± 0.08	1.27 ± 0.06	1.27 ± 0.08	1.21 ± 0.09	0.82 ± 0.08	1.53 ± 0.10	-	0.74 ± 0.10	1.22 ± 0.04
**IL-1β**	0.63 ± 0.06	0.96 ± 0.09	0.89 ± 0.04	1.25 ± 0.04	0.69 ± 0.02	1.09 ± 0.04	1.86 ± 0.02	0.87 ± 0.04	0.98 ± 0.02
**IL-2**	0.61 ± 0.14	0.96 ± 0.13	0.89 ± 0.05	0.63 ± 0.04	0.74 ± 0.02	1.16 ± 0.03	1.39 ± 0.04	0.72 ± 0.06	0.87 ± 0.02
**IL-6**	0.55 ± 0.02	0.88 ± 0.02	0.89 ± 0.04	0.90 ± 0.02	0.78 ± 0.04	1.03 ± 0.02	1.77 ± 0.04	0.66 ± 0.02	1.33 ± 0.04
**IL-8**	0.42 ± 0.02	1.04 ± 0.02	0.87 ± 0.02	0.87 ± 0.02	0.69 ± 0.02	1.06 ± 0.02	1.97 ± 0.04	0.70 ± 0.02	0.89 ± 0.02

**Table 4:** MT1-MMP, MMP-11 and 18S rRNA Target:Control ratios for 3, 6 and 12 hour treatments of MEF cells.

**Treatment**	**Target:Control Ratio**								
	**3 Hours**			**6 Hours**			**12 Hours**		
	
	MT1-MMP ± SEM	MMP-11 ± SEM	18S rRNA ± SEM	MT1-MMP ± SEM	MMP-11 ± SEM	18S rRNA ± SEM	MT1-MMP ± SEM	MMP-11 ± SEM	18S rRNA ± SEM

**Control**	1.00 ± 0.06	1.00 ± 0.14	1.00 ± 0.04	1.00 ± 0.08	1.00 ± 0.10	1.00 ± 0.04	1.00 ± 0.08	1.00 ± 0.08	1.00 ± 0.02
**TGF-β**	0.52 ± 0.06	1.14 ± 0.08	1.35 ± 0.14	0.96 ± 0.08	1.14 ± 0.06	1.35 ± 0.16	0.80 ± 0.04	0.95 ± 0.08	1.34 ± 0.12
**IL-1β**	0.58 ± 0.06	0.74 ± 0.04	1.10 ± 0.10	1.12 ± 0.10	0.74 ± 0.06	1.24 ± 0.08	0.70 ± 0.04	0.75 ± 0.08	1.10 ± 0.08
**IL-2**	0.75 ± 0.14	0.90 ± 0.08	0.94 ± 0.06	0.59 ± 0.02	0.68 ± 0.05	0.95 ± 0.06	0.70 ± 0.04	0.75 ± 0.04	0.90 ± 0.02
**IL-6**	0.56 ± 0.02	0.90 ± 0.02	0.75 ± 0.06	0.85 ± 0.05	0.78 ± 0.08	1.15 ± 0.07	0.64 ± 0.02	0.74 ± 0.06	1.20 ± 0.02
**IL-8**	0.45 ± 0.03	0.96 ± 0.04	0.90 ± 0.02	0.75 ± 0.05	0.74 ± 0.05	1.00 ± 0.08	0.92 ± 0.04	0.76 ± 0.03	0.92 ± 0.02

**Table 5:** MT1-MMP, MMP-11 and 18S rRNA Target:Control ratios for 24 and 48 hour treatments of NIH3T3 cells.

**Treatment**	**Target:Control Ratio**					
	**24 Hour**			**48 Hour**		
	
	MT1-MMP ± SEM	MMP-11 ± SEM	18S rRNA ± SEM	MT1-MMP ± SEM	MMP-11 ± SEM	18S rRNA ± SEM

**Control**	1.00 ± 0.02	1.00 ± 0.02	1.00 ± 0.02	1.00 ± 0.08	1.00 ± 0.04	1.00 ± 0.05
**Collagen I**	1.00 ± 0.08	1.35 ± 0.10	0.90 ± 0.08	0.59 ± 0.06	1.27 ± 0.08	0.96 ± 0.04
**Collagen IV**	0.60 ± 0.06	0.68 ± 0.08	0.58 ± 0.06	0.35 ± 0.10	0.71 ± 0.10	0.60 ± 0.05
**Collagen V**	1.10 ± 0.10	1.65 ± 0.08	0.60 ± 0.06	0.96 ± .004	1.91 ± 0.08	0.56 ± 0.08
**Con-A**	1.00 ± 0.14	1.35 ± 0.12	0.78 ± 0.08	0.82 ± 0.10	1.41 ± 0.14	0.80 ± 0.02
**TGF-β**	0.95 ± 0.06	0.56 ± 0.06	0.60 ± 0.02	0.70 ± 0.12	1.67 ± 0.14	0.56 ± 0.10
**Fibronectin**	1.18 ± 0.12	0.72 ± 0.08	0.58 ± 0.02	0.95 ± 0.32	2.16 ± 0.24	0.52 ± 0.01
**IL-1β**	1.14 ± 0.02	0.78 ± 0.04	0.55 ± 0.02	1.09 ± 0.18	2.20 ± 0.24	0.48 ± 0.02
**IL-2**	1.33 ± 0.20	0.70 ± 0.08	0.50 ± 0.02	1.01 ± 0.02	1.57 ± 0.16	0.48 ± 0.02
**IL-6**	0.80 ± 0.04	0.74 ± 0.04	0.65 ± 0.02	0.75 ± 0.06	1.27 ± 0.08	0.68 ± 0.04

**Table 6:** MT1-MMP, MMP-11 and 18S rRNA Target:Control ratios for 24 and 48 hour treatments of MEF cells.

**Treatment**	**Target:Control Ratio**					
	**24 Hour**			**48 Hour**		
	
	MT1-MMP ± SEM	MMP-11 ± SEM	18S rRNA ± SEM	MT1-MMP ± SEM	MMP-11 ± SEM	18S rRNA ± SEM

**Control**	1.00 ± 0.02	1.00 ± 0.02	1.00 ± 0.02	1.00 ± 0.08	1.00 ± 0.04	1.00 ± 0.05
**Collagen I**	1.00 ± 0.08	1.38 ± 0.18	0.90 ± 0.04	0.84 ± 0.06	1.20 ± 0.10	0.94 ± 0.04
**Collagen IV**	1.10 ± 0.06	1.65 ± 0.08	0.58 ± 0.06	1.00 ± 0.08	1.45 ± 0.12	0.60 ± 0.05
**Collagen V**	1.04 ± 0.10	1.65 ± 0.08	0.60 ± 0.06	0.90 ± .004	1.80 ± 0.10	0.56 ± 0.02
**Con-A**	1.20 ± 0.14	0.66 ± 0.12	0.78 ± 0.08	0.95 ± 0.08	0.85 ± 0.10	0.80 ± 0.04
**TGF-β**	0.90 ± 0.06	0.56 ± 0.06	0.60 ± 0.02	0.84 ± 0.08	0.75 ± 0.05	0.64 ± 0.08
**Fibronectin**	1.07 ± 0.12	0.71 ± 0.08	0.58 ± 0.02	0.95 ± 0.32	0.95 ± 0.07	0.64 ± 0.04
**IL-1β**	1.34 ± 0.02	0.65 ± 0.04	0.55 ± 0.02	1.45 ± 0.16	1.86 ± 0.20	0.55 ± 0.03
**IL-2**	0.88 ± 0.20	0.66 ± 0.08	0.50 ± 0.02	0.96 ± 0.04	1.50 ± 0.10	0.56 ± 0.06
**IL-6**	1.03 ± 0.04	0.72 ± 0.04	0.65 ± 0.02	0.75 ± 0.06	1.15 ± 0.12	0.60 ± 0.02

**Table 7:** MT1-MMP and MMP-11 Target:Control ratios for 24 and 48 hour treatments normalised to 18S rRNA of NIH3T3 cells.

**Treatment**	**Target:Control Ratio**			
	**24 Hour**		**48 Hour**	
	
	MT1-MMP ± SEM	MMP-11 ± SEM	MT1-MMP ± SEM	MMP-11 ± SEM

**Control**	1.00 ± 0.02	1.00 ± 0.02	1.00 ± 0.08	1.00 ± 0.04
**Collagen I**	1.32 ± 0.08	1.35 ± 0.10	1.32 ± 0.08	1.27 ± 0.08
**Collagen IV**	1.26 ± 0.06	0.68 ± 0.08	1.26 ± 0.06	0.71 ± 0.10
**Collagen V**	1.60 ± 0.08	1.65 ± 0.08	3.55 ± 0.35	1.91 ± 0.08
**Con-A**	1.00 ± 0.02	1.35 ± 0.12	1.00 ± 0.02	1.41 ± 0.14
**TGF-β**	0.62 ± 0.04	0.56 ± 0.06	2.95 ± 0.25	1.67 ± 0.14
**Fibronectin**	0.75 ± 0.08	0.72 ± 0.08	4.65 ± 0.38	2.16 ± 0.24
**IL-1β**	0.72 ± 0.10	0.78 ± 0.04	4.32 ± 0.28	2.20 ± 0.24
**IL-2**	0.68 ± 0.08	0.70 ± 0.08	3.36 ± 0.30	1.57 ± 0.16
**IL-6**	0.70 ± 0.04	0.74 ± 0.04	1.90 ± 0.14	1.27 ± 0.08

**Table 8:** MT1-MMP and MMP-11 Target:Control ratios for 24 and 48 hour treatments normalised to 18S rRNA of NIH3T3 cells of MEF cells.

**Treatment**	**Target:Control Ratio**			
	**24 Hour**		**48 Hour**	
	
	MT1-MMP ± SEM	MMP-11 ± SEM	MT1-MMP ± SEM	MMP-11 ± SEM

**Control**	1.00 ± 0.02	1.00 ± 0.02	1.00 ± 0.08	1.00 ± 0.04
**Collagen I**	1.28 ± 0.04	1.30 ± 0.6	1.28 ± 0.08	1.32 ± 0.04
**Collagen IV**	1.35 ± 0.05	0.76 ± 0.04	1.35 ± 0.05	0.68 ± 0.02
**Collagen V**	1.54 ± 0.02	1.45 ± 0.10	2.85 ± 0.35	1.68 ± 0.04
**Con-A**	1.00 ± 0.02	1.26 ± 0.06	1.00 ± 0.02	1.35 ± 0.05
**TGF-β**	0.75 ± 0.05	0.65 ± 0.03	2.60 ± 0.18	1.44 ± 0.12
**Fibronectin**	0.85 ± 0.05	0.90 ± 0.04	4.20 ± 0.26	1.86 ± 0.18
**IL-1β**	0.68 ± 0.08	0.70 ± 0.04	4.00 ± 0.20	1.95 ± 0.17
**IL-2**	0.74 ± 0.06	0.68 ± 0.02	3.14 ± 0.26	1.44 ± 0.10
**IL-6**	0.70 ± 0.02	0.80 ± 0.05	1.74 ± 0.12	1.15 ± 0.07

**Table 9:** MT1-MMP and MMP-11 Target:Control ratios for co-cultures with HBC cell lines normalised to 18S rRNA of NIH3T3 cells and MEF cells.

**Treatment**	**Target:Control Ratio**			
	**NIH3T3**		**MEF**	
	
	MT1-MMP ± SEM	MMP-11 ± SEM	MT1-MMP ± SEM	MMP-11 ± SEM

**0.5 × Control**	0.73 ± 0.08	0.40 ± 0.13	0.65 ± 0.10	0.38 ± 0.10
**2.0 × Control**	1.37 ± 0.18	1.48 ± 0.37	1.24 ± 0.08	1.55 ± 0.23
**Control**	1.00 ± 0.06	1.00 ± 0.09	1.00 ± 0.06	1.00 ± 0.09
**MDA-MB-231**	0.81 ± 0.08	0.71 ± 0.18	0.87 ± 0.09	0.65 ± 0.13
**MDA-MB-436**	1.04 ± 0.02	0.76 ± 0.11	1.14 ± 0.08	0.70 ± 0.08
**MCF-7**	0.82 ± 0.09	0.64 ± 0.31	0.86 ± 0.10	0.72 ± 0.26
**MDA-MB-435**	0.99 ± 0.11	0.65 ± 0.12	1.00 ± 0.13	0.62 ± 0.08
**ML-20**	1.22 ± 0.12	1.21 ± 0.27	1.16 ± 0.06	1.33 ± 0.15
